# Supplementary material for: The Real-World Burden of Moderate-to-Severe Psoriasis in Patients Under Systemic Treatment from Baltic Countries: Data from the CRYSTAL Observational Study
Source: Medicina (Kaunas). 2025 Feb 25;61(3):397. doi: 10.3390/medicina61030397 (PMC11943988; doi:10.3390/medicina61030397)
Supplement: Supplementary file 1 [file medicina-61-00397-s001.zip › medicina-3442722-supplementary.pdf]

Table S1. Distribution of patients in each country by PASI score at study visit, overall, and by current systemic treatment (full analysis set).

**Characteristic**	**n (%)**		
	**Estonia**	**Latvia**	**Lithuania**
Overall			
PASI score ≤1	28 (56.0%)	24 (48.0%)	14 (28.0%)
PASI score ≤3	41 (82.0%)	35 (70.0%)	32 (64.0%)
PASI score ≤5	48 (96.0%)	38 (76.0%)	37 (74.0%)
PASI score >5–≤10	1 (2.0%)	4 (8.0%)	8 (16.0%)
PASI score >10–≤20	1 (2.0%)	6 (12.0%)	4 (8.0%)
PASI score >20	0 (0.0%)	2 (4.0%)	1 (2.0%)
Monotherapy			
PASI score ≤1	26 (56.5%)	24 (48.0%)	9 (25.0%)
PASI score ≤3	37 (80.4%)	35 (70.0%)	22 (61.1%)
PASI score ≤5	44 (95.7%)	38 (76.0%)	26 (72.2%)
PASI score >5–≤10	1 (2.2%)	4 (8.0%)	6 (16.7%)
PASI score >10–≤20	1 (2.2%)	6 (12.0%)	3 (8.3%)
PASI score >20	0 (0.0%)	2 (4.0%)	1 (2.8%)
Monotherapy with biological agents			
PASI score ≤1	26 (66.7%)	24 (48.0%)	9 (26.5%)
PASI score ≤3	35 (89.7%)	35 (70.0%)	21 (61.8%)
PASI score ≤5	38 (97.4%)	38 (76.0%)	25 (73.5%)
PASI score >5–≤10	1 (2.6%)	4 (8.0%)	6 (17.6%)
PASI score >10–≤20	0 (0.0%)	6 (12.0%)	2 (5.9%)
PASI score >20	0 (0.0%)	2 (4.0%)	1 (2.9%)
Monotherapy with conventional agents			
PASI score ≤1	7 (100.0%)	-	2 (100.0%)
PASI score ≤3	2 (28.6%)	-	1 (50.0%)
PASI score ≤5	6 (85.7%)	-	1 (50.0%)
PASI score >5–≤10	0 (0.0%)	-	0 (0.0%)
PASI score >10–≤20	1 (14.3%)	-	1 (50.0%)
PASI score >20	0 (0.0%)	-	0 (0.0%)
Combination therapy			
PASI score ≤1	2 (50.0%)	-	5 (35.7%)
PASI score ≤3	4 (100.0%)	-	10 (71.4%)
PASI score ≤5	4 (100.0%)	-	11 (78.6%)
PASI score >5–≤10	0 (0.0%)	-	2 (14.3%)
PASI score >10–≤20	0 (0.0%)	-	1 (7.1%)
PASI score >20	0 (0.0%)	-	0 (0.0%)

n (%): number (percentage) of patients in each category; PASI: Psoriasis Area and Severity Index.

Table S2. WPAI-PSO domain scores by absolute PASI scores at study visit in each country (full analysis set).

	Estonia				Latvia				Lithuania			
	n	n_{miss}	Mean (SD)	Median (IQR)	n	n_{miss}	Mean (SD)	Median (IQR)	n	n_{miss}	Mean (SD)	Median (IQR)
**Overall**												
$N/N_{employed}$			50/41				50/41				50/36	
Absenteeism	38	3	1.4 (5.3)	0.0 (0.0–0.0)	39	2	0.6 (2.6)	0.0 (0.0–0.0)	36	0	4.6 (13.8)	0.0 (0.0–0.0)
Presenteeism	38	3	3.2 (7.7)	0.0 (0.0–0.0)	39	2	7.2 (14.3)	0.0 (0.0–10.0)	36	0	10.6 (19.7)	0.0 (0.0–15.0)
Work productivity loss	38	3	4.2 (11.2)	0.0 (0.0–0.0)	39	2	7.7 (14.8)	0.0 (0.0–10.0)	36	0	12.8 (22.8)	0.0 (0.0–18.3)
Activity impairment	50	0	6.6 (16.9)	0.0 (0.0–0.0)	50	0	11.6 (21.0)	0.0 (0.0–20.0)	50	0	14.4 (19.3)	0.0 (0.0–30.0)
**Subpopulation with PASI ≤1 at study visit**												
$N/N_{employed}$			28/23				24/20				14/9	
Absenteeism	21	2	0.4 (1.9)	0.0 (0.0–0.0)	20	0	0.2 (0.7)	0.0 (0.0–0.0)	9	0	2.2 (5.5)	0.0 (0.0–0.0)
Presenteeism	21	2	1.9 (5.1)	0.0 (0.0–0.0)	20	0	4.5 (12.3)	0.0 (0.0–0.0)	9	0	0.0 (0.0)	0.0 (0.0–0.0)
Work productivity loss	21	2	2.2 (6.4)	0.0 (0.0–0.0)	20	0	4.6 (12.5)	0.0 (0.0–0.0)	9	0	2.2 (5.5)	0.0 (0.0–0.0)
Activity impairment	28	0	4.6 (13.5)	0.0 (0.0–0.0)	24	0	5.8 (12.8)	0.0 (0.0–0.0)	14	0	2.9 (10.7)	0.0 (0.0–0.0)
**Subpopulation with PASI >1 at study visit**												
$N/N_{employed}$			22/18				26/21				36/27	
Absenteeism	17	1	2.6 (7.5)	0.0 (0.0–0.0)	19	2	1.1 (3.6)	0.0 (0.0–0.0)	27	0	5.4 (15.6)	0.0 (0.0–0.0)
Presenteeism	17	1	4.7 (10.1)	0.0 (0.0–0.0)	19	2	10.0 (16.0)	0.0 (0.0–20.0)	27	0	14.1 (21.7)	0.0 (0.0–20.0)
Work productivity loss	17	1	6.6 (15.1)	0.0 (0.0–0.0)	19	2	10.9 (16.7)	0.0 (0.0–20.0)	27	0	16.4 (25.3)	2.4 (0.0–25.0)
Activity impairment	22	0	9.1 (20.4)	0.0 (0.0–0.0)	26	0	16.9 (25.6)	0.0 (0.0–30.0)	36	0	18.9 (20.1)	15.0 (0.0–30.0)
**Subpopulation with PASI ≤3 at study visit**												
$N/N_{employed}$			41/33				35/30				32/24	
Absenteeism	31	2	1.1 (4.7)	0.0 (0.0–0.0)	29	1	0.3 (1.1)	0.0 (0.0–0.0)	24	0	0.8 (3.4)	0.0 (0.0–0.0)
Presenteeism	31	2	2.6 (6.8)	0.0 (0.0–0.0)	29	1	6.9 (15.4)	0.0 (0.0–0.0)	24	0	5.8 (12.5)	0.0 (0.0–5.0)
Work productivity loss	31	2	3.4 (9.9)	0.0 (0.0–0.0)	29	1	7.1 (15.5)	0.0 (0.0–0.0)	24	0	6.6 (12.6)	0.0 (0.0–10.0)
Activity impairment	41	0	4.1 (12.6)	0.0 (0.0–0.0)	35	0	7.1 (16.9)	0.0 (0.0–0.0)	32	0	8.1 (15.1)	0.0 (0.0–15.0)

**Subpopulation with PASI >3 at study visit**												
$N/N_{employed}$			9/8				15/11				18/12	
Absenteeism	7	1	2.9 (7.6)	0.0 (0.0–0.0)	10	1	1.5 (4.8)	0.0 (0.0–0.0)	12	0	12.1 (22.0)	1.2 (0.0–11.5)
Presenteeism	7	1	5.7 (11.3)	0.0 (0.0–10.0)	10	1	8.0 (11.4)	0.0 (0.0–20.0)	12	0	20.0 (27.6)	10.0 (0.0–30.0)
Work productivity loss	7	1	7.7 (16.4)	0.0 (0.0–10.0)	10	1	9.2 (13.3)	0.0 (0.0–20.0)	12	0	25.2 (32.8)	12.1 (0.0–36.7)
Activity impairment	9	0	17.8 (27.7)	0.0 (0.0–30.0)	15	0	22.0 (26.2)	20.0 (0.0–30.0)	18	0	25.6 (21.2)	25.0 (10.0–40.0)
**Subpopulation with PASI ≤5 at study visit**												
$N/N_{employed}$			48/40				38/33				37/26	
Absenteeism	37	3	1.4 (5.3)	0.0 (0.0–0.0)	32	1	0.3 (1.0)	0.0 (0.0–0.0)	26	0	0.7 (3.3)	0.0 (0.0–0.0)
Presenteeism	37	3	3.2 (7.8)	0.0 (0.0–0.0)	32	1	6.6 (14.7)	0.0 (0.0–5.0)	26	0	6.9 (13.8)	0.0 (0.0–10.0)
Work productivity loss	37	3	4.3 (11.3)	0.0 (0.0–0.0)	32	1	6.8 (14.9)	0.0 (0.0–5.0)	26	0	7.7 (13.8)	0.0 (0.0–10.0)
Activity impairment	48	0	4.6 (12.9)	0.0 (0.0–0.0)	38	0	6.8 (16.3)	0.0 (0.0–0.0)	37	0	11.1 (17.4)	0.0 (0.0–20.0)
**Subpopulation with PASI >5 at study visit**												
$N/N_{employed}$			2/1				12/8				13/10	
Absenteeism	1	0	0.0 (.)	0.0 (0.0–0.0)	7	1	2.2 (5.7)	0.0 (0.0–0.0)	10	0	14.5 (23.5)	3.6 (0.0–16.7)
Presenteeism	1	0	0.0 (.)	0.0 (0.0–0.0)	7	1	10.0 (12.9)	0.0 (0.0–20.0)	10	0	20.0 (29.1)	10.0 (0.0–20.0)
Work productivity loss	1	0	0.0 (.)	0.0 (0.0–0.0)	7	1	11.7 (15.1)	0.0 (0.0–30.0)	10	0	26.3 (35.0)	12.1 (0.0–33.3)
Activity impairment	2	0	55.0 (35.4)	55.0 (30.0–80.0)	12	0	26.7 (27.4)	25.0 (0.0–35.0)	13	0	23.8 (21.8)	20.0 (10.0–40.0)

IQR: interquartile range; N: number of participants who completed the questionnaires; n_{miss}: number of patients with missing data; n (%): number (percentage) of patients in each category; PASI: Psoriasis Area and Severity Index; SD: standard deviation; WPAI-PSO: Work Productivity and Activity Impairment Questionnaire for Psoriasis.

Note: WPAI-PSO questionnaires were filled in only by employed participants for the absenteeism, presenteeism, and work productivity loss domain, and by all participants for the activity impairment domain.

File S1. Scoring of physician assessments and patient-reported outcomes.

A1. Psoriasis Area and Severity Index (PASI) component scores

The PASI combines the assessment of lesion severity and the affected area into a total score ranging from 0 (no disease) to 72 (maximal disease). The PASI measures the intensity of redness, thickness, and scaliness of the lesions (each graded as 0 [none], 1 [mild], 2 [moderate], 3 [severe], or 4 [very severe]) weighted by the percentage area of involvement (graded as 0 [0%], 1 [1–9%], 2 [10–29%], 3 [30–49%], 4 [50–69%], 5 [70–89%], or 6 [90–100%]) in each of the four regions (components) of the patient: head and neck, upper extremities, trunk, and lower extremities (1).

The PASI score was calculated as follows for each of the four PASI components:

- Head and neck: A_1*B_1*0.1;
- Upper extremities: A_2*B_2*0.2;
- Trunk: A_3*B_3*0.3;
- Lower extremities: A_4*B_4*0.4.

In the calculations, A_i is the sum of the three intensity scores and B_i is the affected area score for each body region.

The total score was calculated as the sum of the four component scores.

A2. Dermatology Life Quality Index (DLQI)

The DLQI consists of a set of ten items/questions (2). Each item is scored on a four-point scale, and a total score of 0–30 is calculated by summing all item scores. All items except for item 7 are scored as 0 (not at all/not relevant), 1 (a little), 2 (a lot), or 3 (very much). Item 7 is scored as 0 (no and a second half of not at all, no with an incomplete second half, or not relevant), 1 (no and a second half of a little), 2 (no and a second half of a lot), or 3 (yes).

The DLQI can be analyzed under six domain scores:

- Symptoms and feelings: sum of scores for items/questions 1 and 2;
- Daily activities: sum of scores for items/questions 3 and 4;
- Leisure: sum of scores for items/questions 5 and 6;
- Work and school: score for item/question 7;
- Personal relationships: sum of scores for items/questions 8 and 9;
- Treatment: score for item/question 10.

If two or more questions were not answered, the total score was not calculated. When calculating domain scores, if the answer to one question was not answered, the score for that domain was not calculated.

A3. EuroQol 5-Dimensions 5-Levels (EQ-5D-5L)

The EQ-5D is a self-completed measure of the patient's health-related quality of life and consists of the EQ-5D descriptive system and the EuroQol-Visual Analogue Scale (EQ-VAS). The EQ-5D-5L descriptive system grades each of five dimensions (mobility, self-care, usual activities, pain/discomfort, and anxiety/depression) across five levels: 1 (no problems), 2 (slight problems), 3 (moderate problems), 4 (severe problems), and 5 (extreme problems).

For the calculation of the EQ-5D utility index score, each patient's responses were assigned to a health state based on value sets provided by the trial sponsor.

A4. Work Productivity and Activity Impairment Questionnaire for Psoriasis (WPAI-PSO)

The WPAI-PSO is a self- or interviewer-administered questionnaire used to assess productivity at work and activity impairment due to a specific health problem (3). The WPAI-PSO evaluates four main outcomes through six questions:

- Absenteeism (percentage of work time missed due to problem for those currently employed): Q2/(Q2+Q4)*100;
- Presenteeism (percentage of impairment while working due to problems for those currently employed and who actually worked in the 7 days prior to the administration of the questionnaire): Q5/10*100;

- Work productivity loss (percentage of overall work impairment due to problems for those currently employed): {Q2/(Q2+Q4) + [(1 – (Q2/Q2+Q4))*(Q5/10)]}*100;
- Activity impairment (percentage of activity impairment due to problems for all responders): Q6/10*100.

For those who missed work and did not actually work in the past 7 days prior to the administration of the questionnaire, the percentage of overall work impairment due to health was equal to the percentage of work time missed due to health.

A5. Patient satisfaction

Patients' satisfaction with the overall control of psoriasis achieved with the current systemic treatment was measured using a single-item seven-point Likert-type scale ranging from satisfied (completely satisfied, mostly satisfied, or somewhat satisfied), through uncertain (either satisfied or dissatisfied), to dissatisfied (somewhat dissatisfied, mostly dissatisfied, or completely dissatisfied).

References

1. Armstrong AW, Parsi K, Schupp CW, Mease PJ, Duffin KC. Standardizing training for psoriasis measures: effectiveness of an online training video on Psoriasis Area and Severity Index assessment by physician and patient raters. *JAMA Dermatol* 2013; 149: 577-582.
2. Finlay AY, Khan GK. Dermatology Life Quality Index (DLQI)--a simple practical measure for routine clinical use. *Clin Exp Dermatol* 1994; 19: 210-216.
3. Reilly Associates. Work Productivity and Activity Impairment Questionnaire (WPAI) Scoring. 2002.

File S2. List of study endpoints included in the analysis by country.

Primary endpoint:

- Mean absolute Psoriasis Area and Severity Index (PASI) score of the overall study population at enrollment.

Secondary endpoints:

- Proportion of patients with an absolute PASI score ≤1, ≤3, and ≤5 at enrollment, overall, and by current systemic treatment option.
- Proportion of patients with an absolute PASI score >5 and >8 at enrollment, overall, and by current systemic treatment option.
- Patient treatment history and systemic treatment type (conventional systemic agents at a chemical substance level, biologics at a drug class level, and oral small-molecule inhibitor of phosphodiesterase-4) used for psoriasis management since disease diagnosis.
- Current systemic treatment by type (monotherapy, combination regimen; conventional systemic agents, biologics, and oral small-molecule inhibitor of phosphodiesterase-4) and chemical substance, treatment duration, starting and current dosage, and dosage intensification(s) since current treatment initiation, including reasons for intensification(s).
- Summary descriptive statistics of demographic and clinical characteristics of interest of patients routinely managed with systemic treatment in the overall study population.
- Summary statistics of Dermatology Life Quality Index (DLQI) total and domain scores at enrollment.
- Proportion of patients with a DLQI total score of 0-1 (i.e., no effect at all of skin disease on patients' health-related quality of life (HRQoL)), 2-5 (i.e., small effect of skin problem on patients' HRQoL), and >5 (i.e., at least a moderate effect on patients' HRQoL) at enrollment, overall, and by current systemic treatment option.
- Summary statistics of EQ-5D-5L utility index score and EuroQol-visual Analogue Scale (EQ-VAS) score at enrollment.
- Proportion of patients with reported problems for each level on each dimension of the EQ-5D and proportion of patients with 'no problems' (i.e., level 1) and 'with problems' (i.e., levels 2 to 5) at enrollment.
- Mean Work Productivity and Activity Impairment Questionnaire for Psoriasis domain scores referring to absenteeism, presenteeism, work productivity loss, and activity impairment in the overall study population and in the different groups of patients by current systemic treatment option and by absolute PASI score at enrollment (i.e., PASI ≤1, ≤3, ≤5, and >5).
- Proportion of patients per satisfaction level as well as of those who were satisfied with the control of their psoriasis with the current treatment (i.e., who scored the Likert scale as somewhat satisfied, mostly satisfied, or completely satisfied) at enrollment.

Exploratory endpoint:

- Mean absolute PASI score at enrollment by current systemic treatment option.
